# Targeting Nanodiamonds to the Nucleus in Yeast Cells

**DOI:** 10.3390/nano10101962

**Published:** 2020-10-02

**Authors:** Aryan Morita, Thamir Hamoh, Alina Sigaeva, Neda Norouzi, Andreas Nagl, Kiran J. van der Laan, Emily P. P. Evans, Romana Schirhagl

**Affiliations:** 1 Department of Biomedical Engineering, University Medical Center Groningen, University of Groningen, Antonius Deusinglaan 1, 9713 AV Groningen, The Netherlands; drg.armorita@gmail.com (A.M.); thamirhamoh@gmail.com (T.H.); aosigaeva@gmail.com (A.S.); n.norouzi2018@gmail.com (N.N.); andreas.nagl@gmail.com (A.N.); kiranvanderlaan@gmail.com (K.J.v.d.L.); e.p.p.evans@student.rug.nl (E.P.P.E.); 2 Department of Dental Biomedical Sciences, Faculty of Dentistry, Universitas Gadjah Mada, Yogyakarta 55281, Indonesia

**Keywords:** fluorescence nanodiamonds, nucleus targeting, yeast cells

## Abstract

Nanodiamonds are widely used for drug delivery, labelling or nanoscale sensing. For all these applications it is highly beneficial to have control over the intracellular location of the particles. For the first time, we have achieved targeting the nucleus of yeast cells. In terms of particle uptake, these cells are challenging due to their rigid cell wall. Thus, we used a spheroplasting protocol to remove the cell wall prior to uptake. To achieve nuclear targeting we used nanodiamonds, which were attached to antibodies. When using non-targeted particles, only 20% end up at the nucleus. In comparison, by using diamonds linked to antibodies, 70% of the diamond particles reach the nucleus.

## 1. Introduction

Fluorescent nanodiamonds (FNDs) show perfectly stable fluorescence [1,2,3]. Thus they are potentially useful for long term fluorescent labelling and tracking [4,5,6]. Additionally, it is particularly useful that FNDs are very well visible for multiple imaging techniques [7]. Furthermore, FNDs are interesting carriers for drugs in drug delivery. Their small size, inertness and biocompatibility leads to longer circulation times or sustained release of the drug [8,9]. Additionally, nanodiamonds can help to dissolve otherwise non-soluble drugs and thus increase bioavailability [10]. Another possible benefit is that nanodiamonds can help drug delivery particles to evade macrophages [11]. Benefits for drug delivery have been demonstrated for a number of drug candidates [12,13]. FNDs also function as a tool for nanoscale intracellular sensing of magnetic fields. They change their optical properties based on their magnetic surrounding. Thus, they have been used to sense temperatures [14,15], pH [16], or magnetic fluctuations [17,18]. They have been introduced to the intracellular environments of many types of cells [19,20] and show excellent biocompatibility [21].

For all the above-mentioned applications, it is essential to not just bring nanodiamonds to random locations, but to control their fate. A limited number of studies have attempted this. In these articles, the nanodiamonds were placed in specific organelles in mammalian cells [22,23,24,25,26]. 

**Figure 1:**
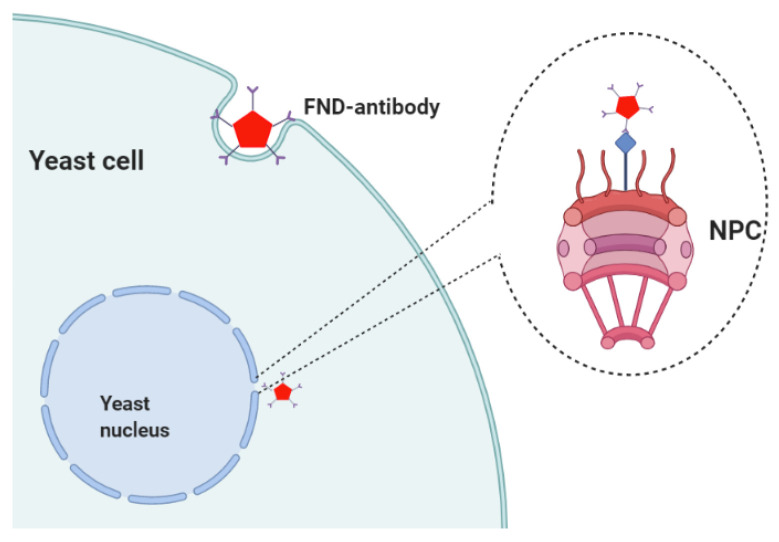
Schematic representation of the targeting strategy in a yeast cell. An antibody with a binding domain for the nuclear pore complex (NPC) was linked to fluorescent nanodiamonds (FNDs). After using a spheroplasting protocol, FNDs–antibodies (FND–AB) are ingested and will accumulate on the nucleus.

Targeting fluorescent nanodiamonds has been done for mostly cancer/tumor diagnostic applications [24,27]. As a first, we have been able to achieve targeting in *Saccharomyces cerevisiae* (yeast cells). For this purpose, we used FNDs linked to antibodies. Linking FNDs to antibodies has already been achieved several times before for different applications [22,28,29]. Yeasts were chosen for this study due to their relevance as a model organism for aging. It is also frequently used in food processing as well as in biotechnology and biosynthesis. Since yeast cells are surrounded by a rigid cell wall, it is important to overcome this barrier. There are several options to achieve this. Firstly, one can either treat yeast cells chemically or with electroporation [19]. This induces pores in the surface or even removes the cell wall altogether [30]. The second process, called spheroplasting, was chosen here because the relatively large particles might be challenging for uptake through small pores. Several different methods exist to achieve spheroplasting [31,32,33]. They might all work for this application, however, we opted for a method that has already been used successfully for nanodiamonds [30]. Within yeast cells, we chose to target the nuclear pore complex (NPC), which is the port of entry to the nucleus. In mammalian cells FND targeting to the nucleus has been achieved before [34]. This is an important organelle since it contains the genetic blue print and plays a key role in the cell cycle [35]. Hence, it is an important target area for drug delivery. In addition to this, DNA damage that occurs in the nucleus causes cancer. This makes the nucleus a compelling location for sensing applications using nanodiamonds. Labelling specific molecules on the nucleus is also of interest for imaging. Lastly, the nucleus is relatively easy to identify which makes it an interesting model system to evaluate and compare targeting strategies. A scheme of the targeting strategy is represented in Figure 1.

## 2. Materials and Methods 

### 2.1. Fluorescent Nanodiamonds Starting Material

Fluorescent nanodiamonds with a hydrodynamic diameter of 70 nm from Adamas Nanotechnology (Releigh, NS, USA) were used for this study. They have a relatively broad size distribution and irregular shape. According to the vendor, these particles are irradiated to host approximately 500 nitrogen vacancy (NV) centers per particle. Since they undergo a cleaning process in oxidizing acid, their surface is oxygen terminated. They have been extensively characterized in previous works [7,36].

### 2.2. Preparation of FNDs Conjugated Antibody

Monoclonal anti-nuclear pore complex (anti-NPC) antibodies (Pro Sci, Poway, CA, USA) were used for preparing antibody conjugated FNDs (FND–AB). According to the vendor, these antibodies have reactivity to yeasts and are produced in mice. Four microliters of anti-NPC antibodies (1 mg/mL) were added to 4 µL of FNDs (1 mg/mL) and were incubated for 20 min at room temperature. We chose to have approximately 7 antibodies per FND. During this process, the antibodies most likely adsorb non-specifically to the diamond surface. Such adsorption has already been reported for a large number of different proteins including antibodies [37,38]. The mixture was vortexed and stored at 4 °C before being used. 

### 2.3. Zeta Potential and Size Measurements

Zeta potential and size measurements of FND–AB were performed with a Malvern Zetasizer Nanosytem (Westborough, MA, USA). The FND–AB were diluted in ultrapure water to a total volume of 1 mL. The samples were measured in triplicate and all the measurements were done at 25 °C. As a control, bare FNDs were measured. To monitor particle behavior in yeast culture medium, zeta potential and size of FND and FND–AB have been measured in a yeast culture medium. 

### 2.4. Fourier-Transform Infrared Spectroscopy (FTIR) Measurements

FTIR spectra were collected to evaluate antibody conjugation to the FND surface and performed with an Agilent FTIR spectrometer (Santa Clara, CA, USA). For this experiment, a germanium crystal was used as a sample holder. After measuring the background of the crystal, 15 µL of FND or FND–AB were dropped onto the crystal and spectra were collected. Further characterization of the particles by optically detected magnetic resonance is shown in the supplementary information, Figure S6. 

### 2.5. Co-Localization of FND–AB

To perform a preliminary evaluation of the conditions needed for antibody conjugation we used fluorescein (FITC) labelled antibodies, which were attached to FNDs. The antibodies we used were monoclonal immunoglobulin G 1 (IgG 1) produced in mice and purchased from ProSci Inc. (Poway, CA, USA). Then, we investigated colocalization. Using this data, we evaluated the stability of the conjugate. If the conjugate was stable, the FITC signal co-localizes with the red signal from FNDs. To perform the assay, the mixture was diluted in ultrapure water and dropped on top of a glass surface. After air-drying, the sample was observed using a Zeiss LSM 780 confocal laser scanning microscope (Zeiss, Jena, Germany). 

**Figure 2:**
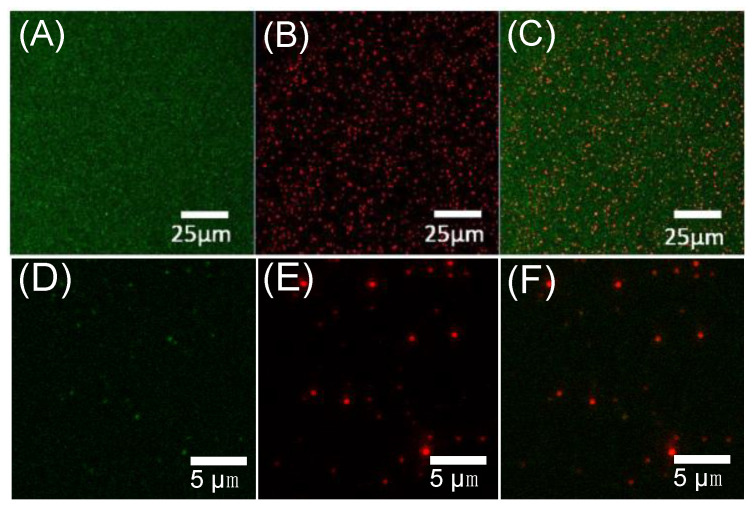
Confocal microscope images of FND–AB. Fluorescein (FITC) labelled antibodies (**A**), FNDs (**B**), and merged images (**C**). (**D**–**F**) show FITC, FND and merged images in magnification.

For FITC labelled antibodies, a 488 nm Argon (Ar) laser with 2% power was used. Since the samples were diluted in water, acquisition was performed between 508–597 nm. The FNDs were imaged using a 561 nm DPSS (diode-pumped solid state) laser (Rossevile, CA, USA), and the photons were collected from 606–694 nm. Identification of FNDs and FITC labelled antibodies was confirmed by measuring spectra in the instrument’s lambda mode. Since the final application of this work is in magnetometry where background light is detrimental, we only used the labelled antibodies for the preliminary evaluation (in Figure 2).

### 2.6. FND Particle Uptake in Saccharomyces Cerevisiae

*Saccharomyces cerevisiae* BY4741 was used in this study. These cells were also used in our previous work where we established the behavior of pristine particles during uptake and cell division [30]. Yeast cells were grown in synthetic dextrose (SD) complete medium until mid-log phase (OD_600_ = 1.05). Since yeast cells have a thick cell wall, it had to be made permeable [19] or removed entirely [30]. Here, we chose to remove the cell wall entirely and form so-called spheroplasts. The spheroplasting protocol was adapted from Karas et al. [39]. To obtain spheroplasts, the cells were washed with sterile demineralized water and centrifuged for 5 min at 2500× *g* at 10 °C. The supernatant was discarded, and 20 mL of 1 M D-sorbitol was added to the cells. The cells were again centrifuged for 5 min at 2500× *g* at 10 °C. After discarding the supernatant, the pellet was diluted in 20 mL of SPEM buffer (consisting of 1 M D-sorbitol, 10 mM EDTA, and 10 mM sodium phosphate) buffer and 40 µL zymolyase 20T (Amsbio, Abingdon, UK). Hereafter, 30 µL of *β*-mercaptoethanol (Sigma, Zwijndrech, The Netherlands) was added. The suspension was then incubated at 30 °C while shaking at 75 rpm for 30 min. Cells were monitored with a light microscope to determine when the spheroplasting process was completed (spheroplasts are brighter than normal yeast cells). The spheroplasting process was stopped by adding 20 mL of 1 M D-sorbitol followed by centrifugation for 5 min at 1000× *g* at 10 °C. After the supernatant was discarded, 2 mL of STC buffer (1 M sorbitol, 10 mM Tris HCl, 10 mM CaCl_2_ and 2.5 mM MgCl_2_) buffer were added to the pellet followed by incubation for 20 min at room temperature. In the end, 50 µL of bare FNDs or FND–AB in 1 M D-sorbitol were added to the suspension followed by 10 min of incubation time at room temperature.

### 2.7. Immobilizing Yeast Cells

In order to monitor single cells, they were immobilized using the following protocol: glass bottom dishes with 4 compartments were coated with 0.1 mg/mL concanavalin A (Sigma, Zwijndrech, The Netherlands) in sterile demineralized water. This coating process was followed by a washing step with sterilized demineralized water. Then the coated dishes were dried in a 37 °C incubator. After drying, 300 µL SD medium without glucose and 4 µL of cell suspension (approximately 2.4 × 10^7^ cells/mL), with internalized particles from the previous step, were added into each quarter.

### 2.8. Biocompatibility Assay 

Biocompatibility of FND and FND–AB has been tested by using an 3-(4,5-dymethylthiazol-2-yl)-2,5-dyphenyltetrazolium bromide (MTT) assay. After creating yeast spheroplasts, cells were placed into 1.5 mL sterile tubes and FND and FND–AB were added into cell suspension. Five milligrams per milliliter of 3-(4,5-dymethylthiazol-2-yl)-2,5-dyphenyltetrazolium bromide (MTT) (Sigma, Zwijndrech, The Netherlands) in sterile PBS was added. Then we incubated at 30 °C for 2 h. Cells were centrifuged at 1000× *g* for 5 min at 10 °C. After discarding the supernatants, 2-propanol (Merck, Amsterdam, The Netherlands) was added to dilute formazan and the samples were vortexed for 5 min. Cells were centrifuged at 1000× *g* for 5 min at 10 °C; then the supernatants were collected and placed into 96-well plates. Absorbance was measured using a microplate reader (Fluostar optima, Ortenberg, Germany) at 540 nm. One percent of H_2_O_2_ (Merck, Amsterdam, The Netherlands) was used as positive control. 

### 2.9. Distance Measurement from the Nucleus

All images were analyzed using FiJi. Z-stacks were recorded starting from the bottom border of the cell to the upper side. By finding the middle layer of the cell with clear cell border and bright particle the images were selected. A scale line was drawn multiple times for statistical purposes between the center of the particle to the border of the nucleus to measure the distance. Cells were chosen randomly. However, we did inspect the cells that were chosen for their size to exclude systematic errors due to this. We did not observe any significant differences in the cell sizes of the respective groups.

### 2.10. Statistical Analysis 

Distance measurements were analyzed using a two-way ANOVA. If the results were significant, a Sidak post hoc test was performed to determine the significance within groups. The results from the MTT assay were analyzed using a one-way ANOVA and followed by Tukey as a post hoc test. Significance level was set at 0.05 and the analysis was performed by using Graph Pad v.8 software (GraphPad Inc, San Diego, CA, USA).

## 3. Results

### 3.1. Characterization of FND–AB 

Characterization of FND–AB was performed by measuring zeta potential and particle size for both groups. We found that the zeta potential of FND–AB was +23.03 ± 0.5 mV while FNDs was −17.87 ± 0.2 mV. Particle size for FND–AB was 523.87 ± 14.8 nm and 83.59 ± 1.2 nm for the FND group, with a particle dispersion index (PDI) of 0.15 ± 0.002 (FND group) and 0.35 ± 0.03 (FND–AB) (see the supplementary information of zeta potential and size distribution graphs, Figures S1 and S2. Additionally, we performed co-localization measurements between fluorescent antibodies and diamonds. From co-localization data, we conclude that approximately 73% of antibodies bind to FNDs. Confocal microscope images of FND–AB are presented in Figure 2.

For the experiments in cells we used non-fluorescent antibodies from which we assume that the binding characteristics to FNDs are comparable. Since free antibodies were invisible in our experiments in cells and did not disturb the targeting (unless they are so abundant that they hinder the conjugated FNDs to bind), there was no need to remove the unreacted antibodies.

### 3.2. Biocompatibility of FND–AB

**Figure 3:**
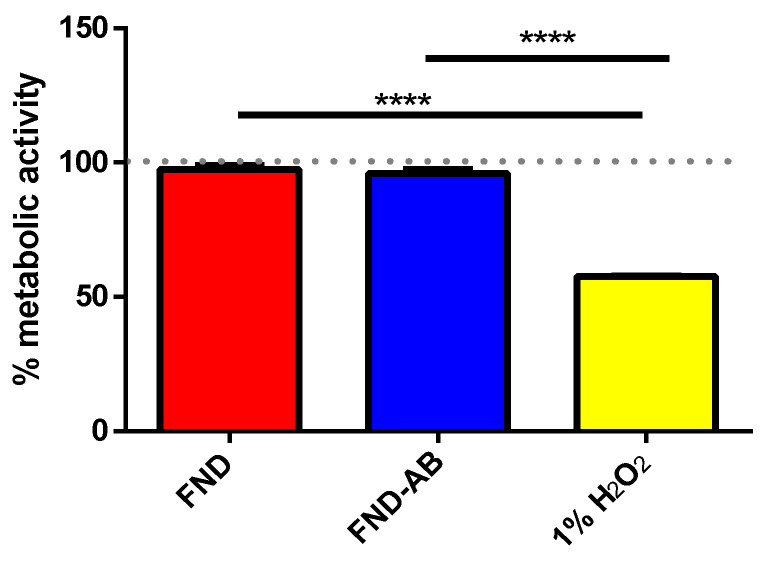
3-(4,5-dymethylthiazol-2-yl)-2,5-dyphenyltetrazolium bromide (MTT) assay for analyzing metabolic activity of yeast cells. Yeasts without any treatment were used as a comparison (grey dots) and 1% of H_2_O_2_ was used as positive control. Compared to yeasts without any treatment, both FND and FND–AB group have no significant effect on metabolic activity in yeast cells. Both groups only show significant difference with the positive control. Data were analyzed by using one-way ANOVA and followed by Tukey post hoc test. The significance level was set at 0.05 and **** indicated *p* < 0.0001.

While the biocompatibility of bare FND has already been shown in the literature, this hasn’t been tested for FND–AB. As expected, the presence of antibody on the FND surface does not have a negative effect on the metabolic activity level of yeasts. Results from MTT assay are presented in Figure 3. Our data also confirms the excellent biocompatibility of FNDs.

### 3.3. Position of FNDs and FND–AB Inside Cells

**Figure 4:**
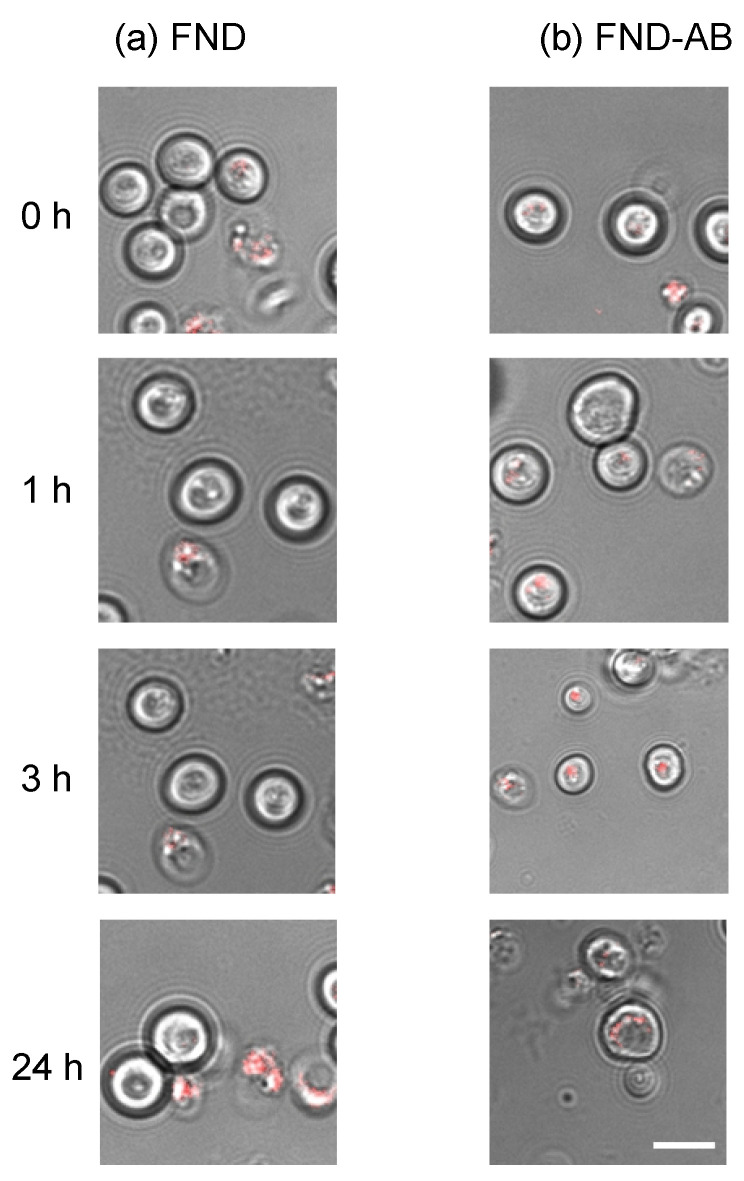
Position of particle (red) inside the cell at different time points. FND (**a**) and FND–AB (**b**) groups were followed between 0 and 24 h after the particles were taken up by the cells. Scale bars in all images are 0.75 µm.

Positions of particles were followed by confocal microscopy between 0 and 24 h after uptake by the cells. It has to be noted here that we did not follow specific cells during 24 h continuously, but that we collected data on the average location. Representative images are shown in Figure 4. Particles in the FND group were mostly located inside the cell, however, they were still only close to the cell wall although they had been followed for 24 h. Contrarily, the particles from the FND–AB group are on average closer to the nucleus with increasing incubation time. The reason is likely that the FND–AB remain attached (longer) to the NPC once they arrive there. It should be noted that there was not much difference in the number of FNDs that were inside the cells. This is due to the excretion of nanodiamonds from yeast over time, which has previously been reported [40].

**Figure 5:**
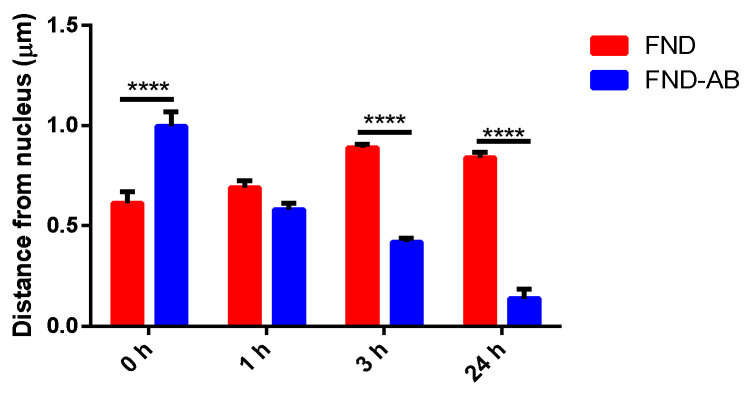
Distance measurement from particles to the border of the nucleus. Experiments were performed in 10 cells. Significance level was set at 0.05. **** indicates *p* < 0.0001. Error bars represent standard errors.

**Figure 6:**
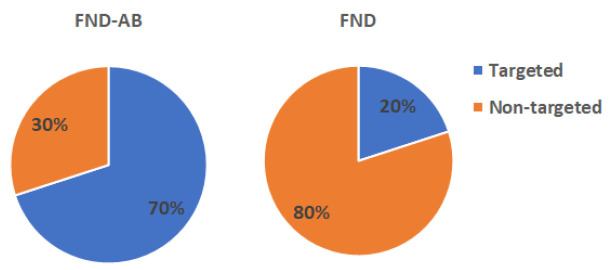
Proportion of particles targeted to the nucleus at the 24 h time point. Fifty particles inside cells were used from both groups.

The successful rate of nanodiamond targeting was evaluated by calculating the distance from the particles to the nucleus (Figure 5), and the proportion of targeted and non-targeted particles from both groups (Figure 6).

According to Figure 4, particles from the FND–AB group were on average closer to the nucleus with increasing incubation time, whilst particles from the FND group remained in the same position. Figure 5 shows that FND–AB were mostly targeted on NPC (70%) compared to FNDs which only had 20% of particles targeted.

## 4. Discussion

We provided a targeting strategy of FNDs in yeast cells using antibodies. Comparing with the literature we can speculate that the antibodies bind primarily via electrostatic interactions. Kong et al. [28] have reported that adsorption of proteins on nanodiamonds generally comes from the interplay of ionic interaction, hydrogen bonding, van der Waals interaction, and hydrophobic interaction between protein and surface. Smith et al. [41] report that primarily hydrostatic interactions play a role in antibody binding. Although their article is on detonation nanodiamonds, this might be the case here as well. We found that using the antibody gave a higher success rate of targeting compared to the bare FND particles. FND–AB were on average closer to the nucleus after the 24 h of incubation (Figure 5). The FNDs, however, remained in the same position (mostly close to the border of the cells (Figure 4)) within the same incubation time. After calculating the proportion of targeted particles (see Figure 6), FND–AB had more cells with targeted particles (70%) compared to the FND group (20%). This means that using a combination of antibody and FND helps to achieve better targeting. 

Targeting nanoparticles to the nucleus has been studied for years. Previous studies mentioned that nuclear targeting for nanoparticles had several barriers like selectivity of cell membrane, endosomal entrapment, lysosomal degradation, cytoplasmic trafficking, and most importantly nuclear entry [42]. According to Chu and co-workers (2015) [43] FNDs have a sharp edged shape which can break the endosomal membrane. It means particles can escape the endosome and degradation of chemical component on the surface (antibody) can be minimized.

Apart from the antibodies we used there are some alternative strategies for targeting. These include controlling nanoparticle size or modifying the surface charge [44]. In this study, using antibodies can modify the surface charge of FND particles. Additionally, since most cell membranes are negatively charged, shifting the surface charge from negative to positive helps cells take up particles more easily. 

In this study, we used FND particles larger than 70 nm. This means the particles could not pass the NPC to enter the nucleus. However, for many applications it is already useful to target the nucleus surface or the NPC.

## 5. Conclusions

Based on the results, we conclude that using antibody linked FNDs helps to achieve intracellular targeting. For further study, controlling the particle size and surface charge might improve the efficacy of targeting FNDs through specific organelles. There are two applications that we foresee for this material. Firstly, the FND–AB could function as long-term fluorescent label. Secondly, and arguably the most interesting, is their possibility to perform nanoscale MRI. The latter concept has been demonstrated earlier this year for random locations [45]. However, the particles demonstrated in this study would allow magnetometry specifically at the nucleus. 

## Supplementary Materials

The following are available online at https://www.mdpi.com/2079-4991/10/10/1962/s1, Figure S1: Average of value of zeta potential from FND and FND-AB particles in water, Figure S2: Average value of particle size distribution from FND and FND-AB in water, Figure S3: Average values of zeta potential from FND and FND-AB in yeast medium, Figure S4: Average value of particle size from FND and FND-AB in yeast medium, Figure S5: FTIR spectra of FND and FND-AB particles, Figure S6: ODMR spectra of FND and FND-AB.
