# Supplementary material for: Targeting Nanodiamonds to the Nucleus in Yeast Cells
Source: Nanomaterials (Basel). 2020 Oct 2;10(10):1962. doi: 10.3390/nano10101962 (PMC7601435; doi:10.3390/nano10101962)
Supplement: Supplementary file 1 [file nanomaterials-10-01962-s001.pdf]

Supplementary information

Targeting Nanodiamonds to the Nucleus in Yeast Cells

Aryan Morita [1,2,†], Thamir Hamoh [1,†], Alina Sigaeva [1], Neda Norouzi [1], Andreas Nagl [1], Kiran J. van der Laan [1], Emily P. P. Evans [1] and Romana Schirhagl [1,*]

[1] Department of Biomedical Engineering, University Medical Center Groningen, University of Groningen, Antonius Deusinglaan 1, 9713 AV Groningen, 9713AV, The Netherlands; drg.armorita@gmail.com (A.M.); thamirhamoh@gmail.com (T.H.); aosigaeva@gmail.com (A.S.); n.norouzi2018@gmail.com (N.N.); andreas.nagl@gmail.com (A.N.); kiranvanderlaan@gmail.com (K.J.v.d.L.); e.p.p.evans@student.rug.nl (E.P.P.E.)

[2] Department of Dental Biomedical Sciences, Faculty of Dentistry, Universitas Gadjah Mada, Yogyakarta, 55281, Indonesia

* Correspondence: romana.schirhagl@gmail.com

† These two authors contributed equally

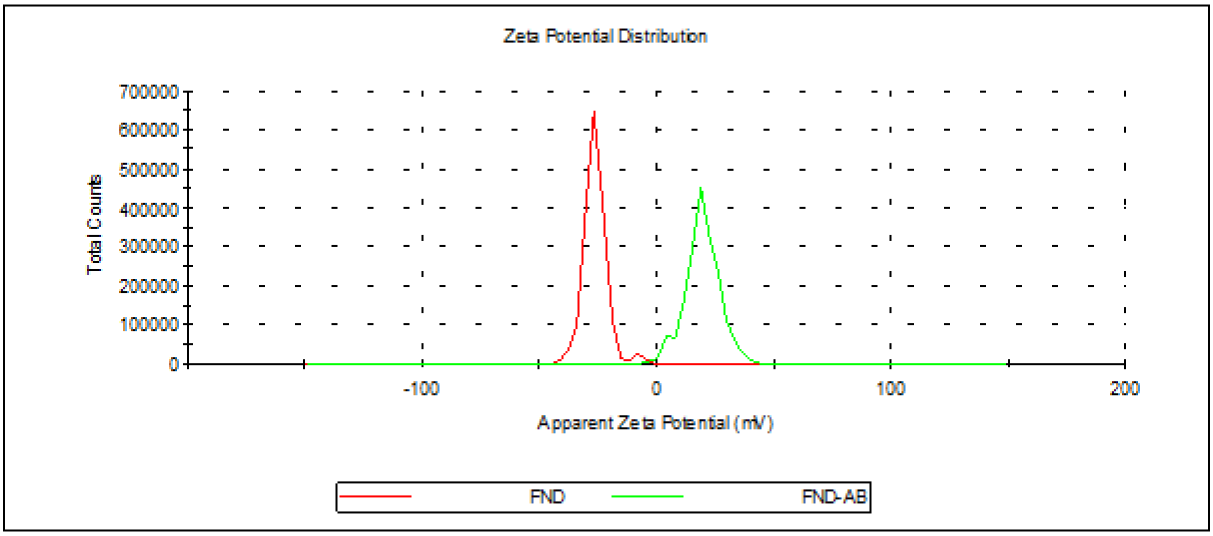

Figure S1. Average of value of value of zeta potential from FND and FND–AB particles in water. FND particles originally have a zeta potential (−17.87 ± 0.2 mV) (left curves) and when they combine with the antibody, the zeta potential becomes positive (+ 23.03 ± 0.5 mV) (right curves). All measurements were performed at 25 °C and in triplicates.

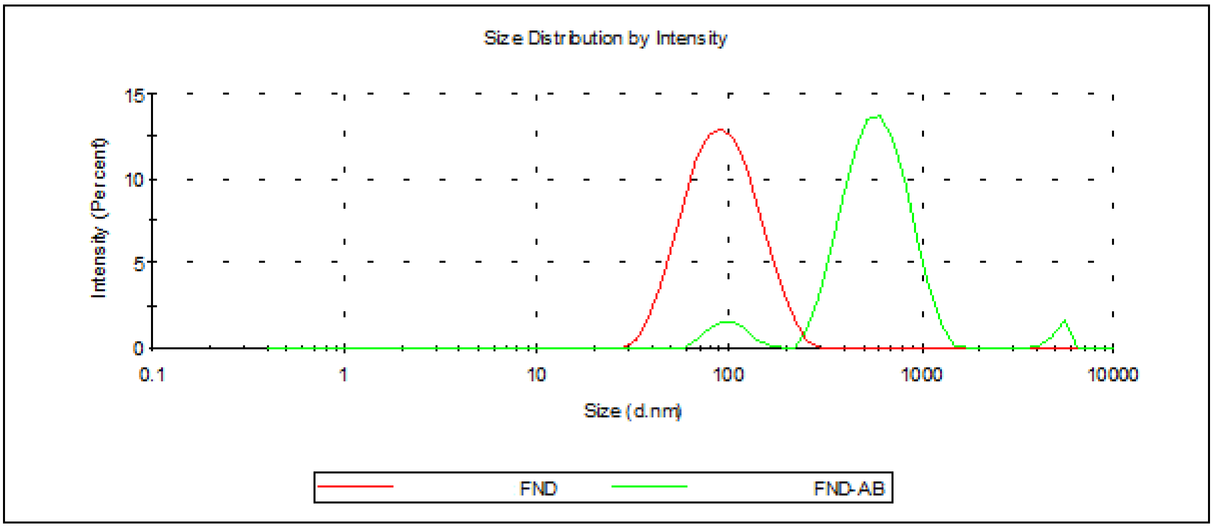

Figure S2. Average value of particle size distribution from FND and FND-AB in water. FND particles have an average size of 83.59 ± 1.2 nm and FND–AB of 523.87 ± 14.8 nm All measurements were performed at 25 °C and in triplicates.

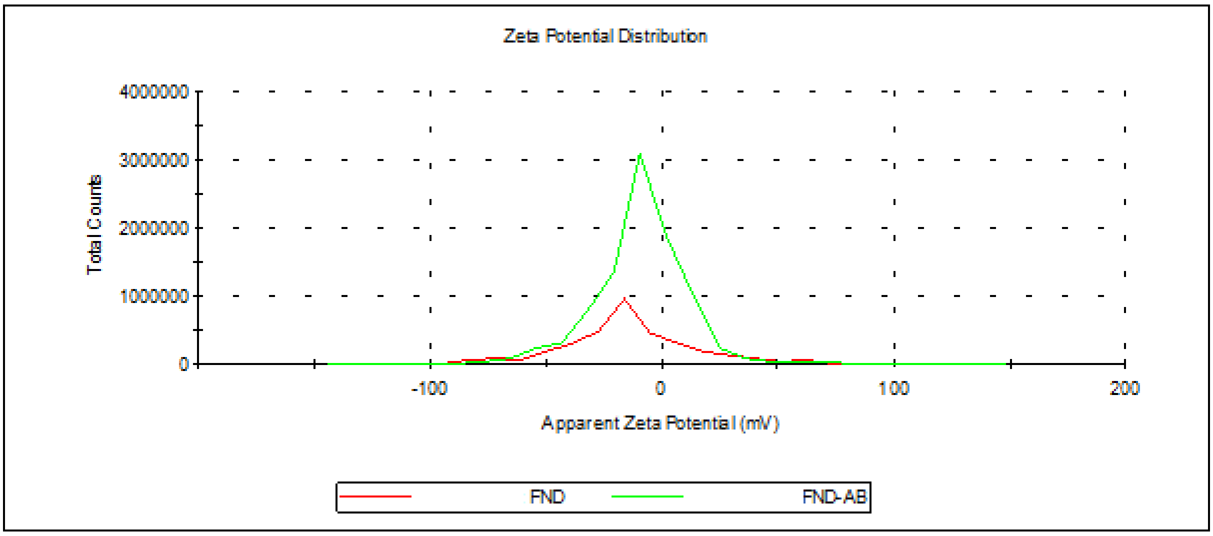

Figure S3. Average values of zeta potential from FND and FND–AB in yeast medium. Yeast medium, which contain yeast nitrogen base and 2% D-Glucose change the zeta potential of FND–AB particles. FND–AB become negative while the FND particles remain negative. All measurement were performed at 25 °C and in triplicates.

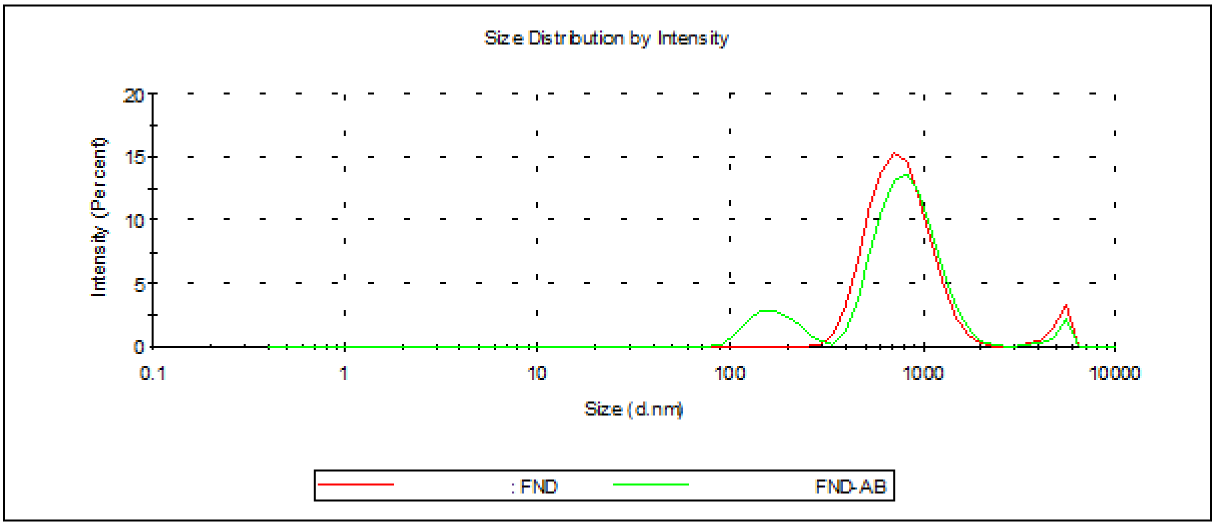

Figure S4. Average value of particle size from FND and FND–AB in yeast medium. Adding both FND and FND–AB into yeast culture medium affects the particle size. Both groups increase drastically in size. All measurements were performed at 25 °C and in triplicates.

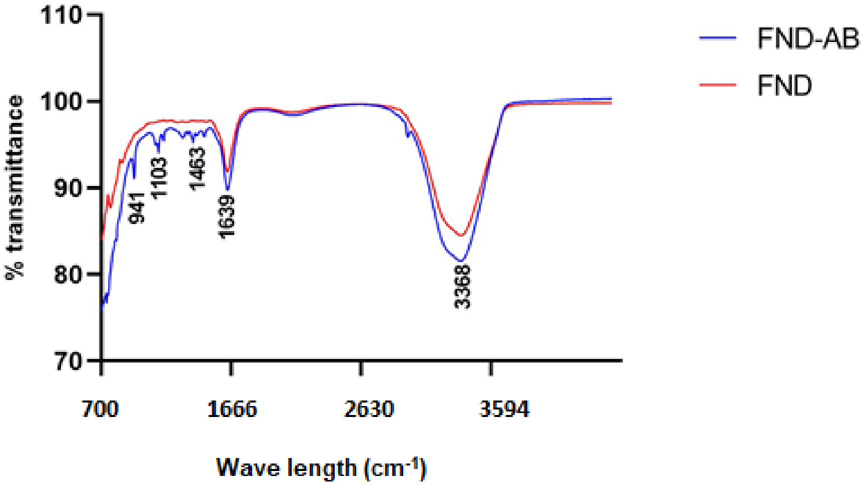

Figure S5. FTIR spectra of FND and FND–AB particles. The difference between the two spectra can be attributed to the presence of antibodies (which are proteins). The broad peak at 3369 cm-1 can be attributed to OH stretching in FND and OH and NH stretching in FND–AB. The small sidepeak at 3300 comes from CH stretching and is absent in the diamond spectrum. The band at 1639 can be attributed to C=C stretching. The band at 1463 comes from CH bending and is also only present in FND-AB. The bands around 1103 are due to CN and CO stretching.

Optically Detected Magnetic Resonance (ODMR) Measurements

ODMR allows to use FNDs to read out their magnetic surrounding, to differentiate nanodiamonds from other particles. After selecting an FND or FND–AB we recorded an optically detected magnetic resonance with a home-built microscope [1]. The frequency was swept around the expected resonance frequency of the NV-centre at 2.87GHz.

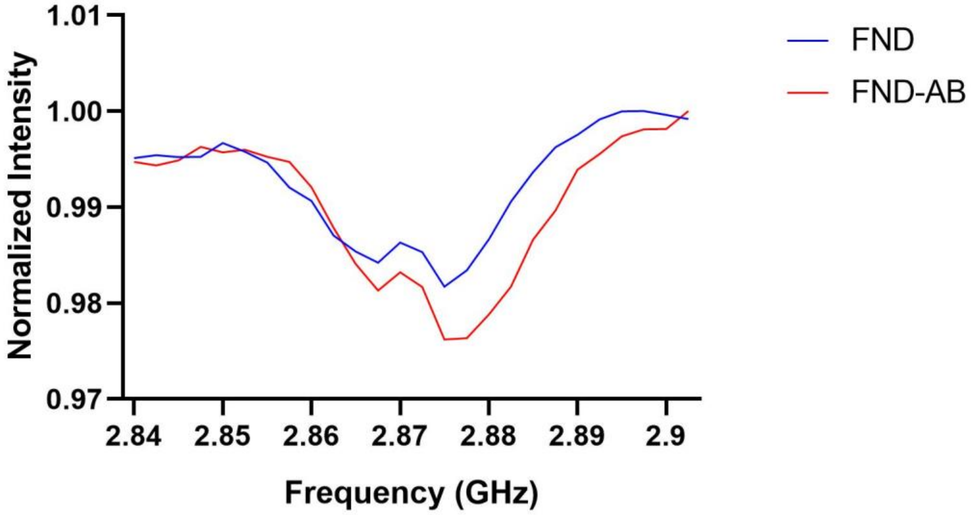

Figure S6. Comparing between ODMR measurements from bare FNDs versus FNDs-antibody, the results are similar. (The difference in intensity is FND dependent and varies with distance from the microwave wire). This confirms that FND–AB are suitable for magnetic sensing experiments.

[1] Hemelaar, S.R., de Boer, P., Chipaux, M. *et al.* Nanodiamonds as multi-purpose labels for microscopy. *Sci Rep* **7,** 720 (2017). https://doi.org/10.1038/s41598-017-00797-2
